# Inoculation of *Sinorhizobium saheli* YH1 Leads to Reduced Metal Uptake for *Leucaena leucocephala* Grown in Mine Tailings and Metal-Polluted Soils

**DOI:** 10.3389/fmicb.2018.01853

**Published:** 2018-08-27

**Authors:** Xia Kang, Xiumei Yu, Yu Zhang, Yongliang Cui, Weiguo Tu, Qiongyao Wang, Yanmei Li, Lanfang Hu, Yunfu Gu, Ke Zhao, Quanju Xiang, Qiang Chen, Menggen Ma, Likou Zou, Xiaoping Zhang, Jinsan Kang

**Affiliations:** ^1^College of Resources, Sichuan Agricultural University, Chengdu, China; ^2^Geomicrobiology Group, School of Life Sciences, University of Dundee, Dundee, United Kingdom; ^3^Sichuan Provincial Academy of Natural Resource and Sciences, Chengdu, China; ^4^Sichuan Earthquake Administration, Chengdu, China

**Keywords:** mine tailings, heavy metals, *Leucaena leucocephala*, *Sinorhizobium*, phytoremediation

## Abstract

Metalliferous mine tailings have a negative impact on the soil environment near mining areas and render cultivable lands infertile. Phytoremediation involving the synergism of legume and rhizobia provides a useful technique in tackling this issue with cost-effective, environmentally friendly, and easy-to-use features under adverse soil conditions. *Leucaena leucocephala* has been found to build symbiotic relationships with native rhizobia in the iron-vanadium-titanium oxide (V-Ti magnetite) mine tailing soil. Rhizobia YH1, isolated from the root nodules of *L. leucocephala*, was classified as *Sinorhizobium saheli* according to similarity and phylogenetic analyses of 16S rRNA, housekeeping and nitrogen fixation genes. Besides nitrogen fixation, *S. saheli* YH1 also showed capabilities to produce indole-acetic acid (IAA) (166.77 ± 2.03 mg l^−1^) and solubilize phosphate (104.41 ± 7.48 mg l^−1^). Pot culture experiments showed that strain YH1 increased the biomass, plant height and root length of *L. leucocephala* by 67.2, 39.5 and 27.2% respectively. There was also an average increase in plant N (10.0%), P (112.2%) and K (25.0%) contents compared to inoculation-free control. The inoculation of YH1 not only reduced the uptake of all metals by *L. leucocephala* in the mine tailings, but also resulted in decreased uptake of Cd by up to 79.9% and Mn by up to 67.6% for plants grown in soils contaminated with Cd/Mn. It was concluded that *S. saheli* YH1 possessed multiple beneficial effects on *L. leucocephala* grown in metalliferous soils. Our findings highlight the role of *S. saheli* YH1 in improving plant health of *L. leucocephala* by reducing metal uptake by plants grown in heavy metal-polluted soils. We also suggest the idea of using *L. leucocephala*-*S. saheli* association for phytoremediation and revegetation of V-Ti mine tailings and soils polluted with Cd or Mn.

## Introduction

Industrial activities, e.g., mining and smelting, are a major source of water and soil pollutions and threaten human health through accumulative effects along food chains (Wuana and Okieimen, 2011). Residents living in proximity to mining areas are continually exposed to hazardous substances released from the factories. Large swathes of cultivable land have been laid to waste as a result of mine tailings being continuously dumped in huge volumes into reservoir-like ponds. Chronic damages to the surrounding soil environment are caused through leaching effects by rainfall. Vanadium-titanium (V-Ti) magnetite mine tailings contain elevated amounts (>2,000 mg kg^−1^) of manganese (Yu et al., 2014), which is considered to be a major metal pollutant in soil and aquatic environments (Li et al., 2014). Cadmium is one of the main heavy metal pollutants that have high cytotoxicity and usually associate with anthropogenic activities such as mining and metal smelting, causing severe contamination to agricultural soils near the vicinity of mines (Liu et al., 2013; Zheng et al., 2018). It is necessary to give a satisfactory solution to this environmental hazard.

Plant growth-promoting rhizobacteria (PGPR) are noted for their capabilities to colonize roots of legumes and at the same time confer beneficial effects on the hosts by alleviating deleterious abiotic stresses (Rajkumar et al., 2010). They have been deemed as a promising approach to the remediation of polluted soils for the rhizobia-legume associations not only promote plant growth but also raise soil nitrogen level, leading to an increased crop yield (Bashan and Holguin, 1998). Several rhizobial species that can form mutualism with legumes are primarily found in genera *Azorhizobium, Bradyrhizobium, Mesorhizobium, Rhizobium, and Sinorhizobium* (Hayat et al., 2010). Symbiotic nitrogen fixers in the rhizosphere colonize root systems through sophisticated mechanisms at both cellular and molecular levels. Legume-rhizobia associations can lead to enhanced plant growth either by biological nitrogen fixation (Sanginga et al., 1988), indole-3-acetic acid (IAA) production, siderophore secretion, and phosphorus solubilization or by a combination of all the above mentioned features (Khan et al., 2009). It was reported that the symbiosis of *Sinorhizobium meliloti* CCNWSX0020 and *Medicago lupulina* exhibited phytostabilizing effects for Cu by boosting plant growth and metal uptake from Cu-spiked soil while decreasing the translocation of Cu in the plant (Kong et al., 2015). In another report, the biofuel legume *Pongamia pinnata* was used for the phytoremediation of V-Ti magnetite mine tailings in partnership with *Bradyrhizobium liaoningense* and both plant growth and metal uptake were significantly increased under multi-metallic conditions (Yu et al., 2016).

*Leucaena leucocephala* first came under notice due to its water holding capacity in a hot and dry climate. Besides, it has strong tolerance and adaptability to drought and therefore is of great importance for agriculture and forestry (Shelton and Brewbaker, 1998). *L. leucocephala* was introduced to Panzhihua city, a major industrial hub in southern Sichuan Province 30 years ago as a pioneer species for afforestation and has ever since been widely cultivated along the hot and arid valleys of Golden Sand River, upper Yangtze (Xu et al., 2013a). A previous study found that it could serve as a pioneer for the revegetation of lead-zinc and tin mine tailings, indicating its potential to thrive under metal-contaminated environments (Li, 2006). In another case, a variant of *L. leucocephala* was discovered to be capable of taking up and metabolizing organic pollutants such as ethylene dibromide and trichloroethylene even without the synergism of rhizobia, which again proves that it could be a potential candidate for the remediation of polluted soils (Doty et al., 2003). Although many other similar studies for the remediation of heavy metal-contaminated sites have been reported of late years, the information on remediation effects of *L. leucocephala* and its associated rhizobia is still scanty.

In the present study, we aim to establish the rhizobia-plant association and explore the feasibility of using it as a novel means for the phytoremediation and revegetation of heavy metal-polluted soils. In this work, we report PGP effects of *Sinorhizobium saheli* YH1 on and reduced metal uptake for *L. leucocephala* in the phytoremediation of both original mine tailings and soils supplemented with Cd or Mn. Our results propound the idea of utilizing the symbiotic system of *L. leucocephala* and *S. saheli* YH1 as an alternative to the alleviation of environmental hazards including Cd, Mn, and other metals.

## Materials and methods

### Sample collection, rhizobia isolation and soil metal measurements

**Figure 1:**
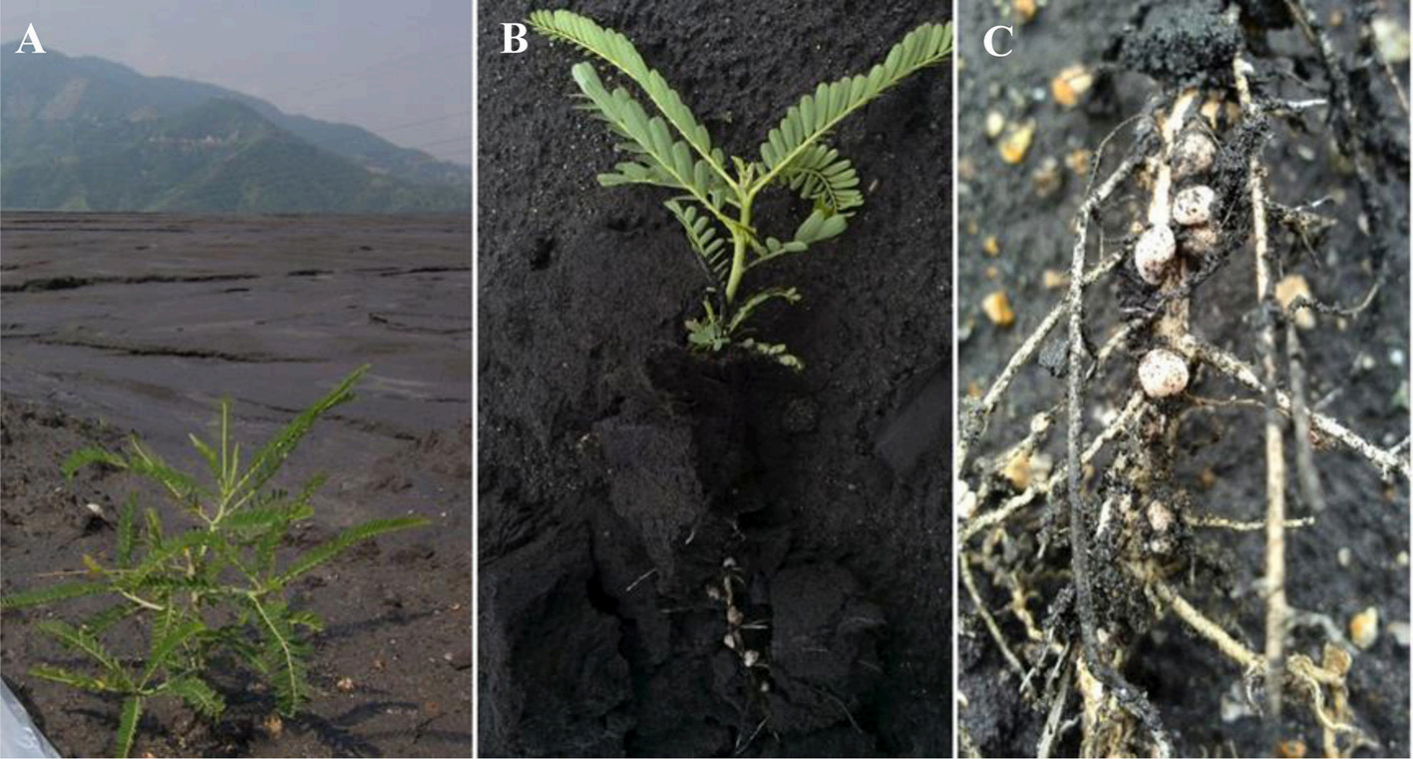
**(A)** Sampling site at Majiatian V-Ti tailings reservoir in Panzhihua mining area and **(B)** a fresh plant sample of *L. leucocephala* with **(C)** vigorously growing root nodules thereon.

Soil samples and *L. leucocephala* seedlings were collected from the edge of a vanadium-titanium magnetite tailing reservoir (Figure 1), situated on the outskirts of Panzhihua city (N26°33′13.23″; E 101°45′59.42″; elevation 1219.3 m). Healthy plants were carefully uprooted, stored in a sterile sampling bag, and shipped back to laboratory. Samples of the V-Ti magnetite tailing soil were taken from the topsoil (0–20 cm deep) within an area of 4 m^2^ consisting of five sampling points near the plants. The isolation of rhizobia was carried out using Congo red-amended yeast mannitol agar (YMA) medium which comprised of (l^−1^ Milli-Q H_2_O): yeast extract 1.0 g, mannitol 10.0 g, K_2_HPO_4_ 0.5 g, MgSO_4_ 0.2 g, NaCl 0.1 g, CaCO_3_ 1.0 g, agar 15.0 g and Congo red 0.025 g (pH 6.8 ± 0.2) (Somasegaran and Hoben, 2012). Fresh root nodules were initially washed with tap water to remove rhizosphere soil. Surface sterilization was performed by soaking root tissues in 75% (v/v) ethanol for 5 min followed by 1% (v/v) sodium hypochlorite for 3 min before they were finally rinsed for 3 times with sterile Milli-Q water (Zhang et al., 2018). Nodules were aseptically cut off from the roots and crushed in a sterile Eppendorf tube with a sterile glass rod. A loopful of the nodule suspension was streaked on a Congo red-containing YMA plate, which was incubated in the dark at 28°C for at least 7 d. Repeated streaking was performed on fresh plates until pure culture was obtained (Somasegaran and Hoben, 2012). Soil samples were thoroughly air-dried at room temperature, put through a sieve (pore size 2 mm). and digested in Teflon crucibles with a concentrated acid mixture of HNO_3_, HF, and HClO_4_ (5:5:3, v/v/v).

### Phylogenetic analyses

Liquid rhizobia culture was used for DNA extraction. The strain was grown in liquid mannitol broth and harvested when cell concentration reached 1 × 10^8^ CFU ml^−1^. Genomic DNA was extracted using TIANamp Bacteria DNA Kit (TIANGEN Biotech, Beijing, China) following standard protocols prescribed by the manufacturer. Fragments of 16S rRNA gene were amplified using the universal primers of 27F (5′-AGA GTT TGA TCC TGG CTC AG-3′) and 1492R (5′-GGT TAC CTT GTT ACG ACT T-3′) (Yu et al., 2014). PCR was performed in a 30 μl buffer system using a Bio-rad T100 Thermo Cycler (Bio-rad, USA) according to the following protocol: an initial denaturation step at 94°C for 3 min, 30 denaturation cycles at 94°C for 1 min, an annealing step at 56°C for 1 min, an extension step at 72°C for 2 min and a final extension at 72°C for 10 min (Tan et al., 1997). The nitrogen fixation gene *nifH* was amplified according to methods described by Laguerre et al. (2001). The amplification of housekeeping genes was carried out using the following primers at specific melting temperatures: atpD255F-atpD782R for *atpD* at Tm 58°C, glnII12F-glnII689R for *glnII* at Tm 56°C, recA63F-recA555R for *recA* at Tm 58°C, and rpoB454F-rpoB1364R for *rpoB* at Tm 65°C (Gaunt et al., 2001; Vinuesa et al., 2005, 2008). PCR products were subjected to electrophoresis in 1% agarose gel to verify the size of target fragments and afterwards sent to Tsing Ke Biological Technology (Chengdu, China) for sequencing. The determined sequences were then compared and aligned with those from known species on GenBank (NCBI, USA) using ClustalW in MEGA 6.0 package (Tamura et al., 2013). Phylogenetic trees were constructed using maximum likelihood method with Kimura 2-parameter (Kimura, 1980) and multi-locus sequence analysis (MLSA) was carried out by constructing a single phylogenetic tree using the concatenated sequences of four housekeeping genes (Tak et al., 2016). The reliability of the phylogenetic trees was calculated based on 1,000 bootstrap repetitions. All sequences mentioned above were deposited in GenBank using BLASTn tool and a unique accession number was assigned to each.

### Metal tolerance tests

The tolerance of rhizobia to cadmium (Cd) and manganese (Mn) was assayed by spot-inoculating cell suspension on yeast mannitol agar. An aliquot of 10 μl initial culture containing 1 × 10^8^ CFU ml^−1^ cells obtained from yeast mannitol broth was pipetted on YMA plates supplemented with 50, 150, 250, and 400 mg l^−1^ of Mn^2+^ or Cd^2+^, respectively. Each treatment had at least three plates as replication and each plate was inoculated with three colonies at equal distance. All plates were maintained in an incubator for 7 d at 28°C in the dark. The metal tolerance was determined by calculating the ratio of the colony size of the experimental group to that of the control.

### Assay of growth promoting traits

Bacterial plant growth promoting abilities including indole-3-acetic acid (IAA) production, siderophore secretion and phosphate solubilization were estimated by measuring the concentrations of IAA, siderophore and solubilized phosphate in liquid media. For the determination of IAA production, rhizobia strains were grown in 5 ml yeast mannitol broth (YMB) supplemented with L-tryptophan (2.5 g l^−1^) at 28°C on a rotary shaker at 150 rpm for 3 days. Culture supernatant was obtained by centrifugation at 8,000 rpm for 5 min. An aliquot of 2 ml supernatant was mixed with 4 ml Salkowski reagent (2% 0.5 M FeCl_3_ in 35% HClO_4_) and immediately incubated in the dark for 30 min at room temperature (Datta and Basu, 2000). The optical density of the mixture after reaction was measured at a wavelength of 530 nm (OD_530_) using a spectrophotometer (WFJ2100, UNICO, China). The actual IAA concentration was calculated in accordance with a standard curve. Siderophore production was estimated using chrome azurol sulfonate (CAS) assay on solid plates according to the method described by Schwyn and Neilands (1987). Liquid National Botanical Research Institute's phosphate medium (NBRIP), which was used to perform quantitative assay of siderophore production, consisted of (l^−1^ Milli-Q H_2_O): MgCl_2_·6H_2_O 5.0 g, MgSO_4_·7H_2_O 0.25 g, KCl 0.2 g, (NH_4_)_2_SO_4_ 0.1 g, Ca_3_(PO_4_)_2_ 5.0 g and glucose 10.0 g. The medium was adjusted to pH 7.0 ± 0.2 before sterilization at 115°C for 15 min. An aliquot of 1 ml bacterial start culture (1 × 10^8^ CFU ml^−1^) was inoculated into a 250 ml Erlenmeyer flask containing 100 ml medium and incubated on a rotary shaker at 150 rpm for 7 days at 28°C (Nautiyal, 1999). The vanadium molybdate blue colorimetric method was employed for the quantification of phosphate, where the absorbance at 660 nm was measured using a spectrophotometer (WFJ2100, UNICO, China) (Walker et al., 2004).

### Pot culture experiment

To assess the effectiveness of the leucaena-rhizobia remediation system, pot culture experiment was carried out in both tailings and metal-spiked soils using Leonard jar apparatus (Somasegaran and Hoben, 2012), which was composed of two parts, i.e., the jar at the bottom (10 × 14 cm) containing plant nutrient solution and the pot (7 × 15 cm) at the top filled with substrate. These two parts were connected by a cotton wick to keep a steady supply of nutrients to the substrate in the pot, and joined together by a sealed screw cap to prevent microbial contamination from the air (Supplementary Figure S1). The jar contained 1 L of nitrogen-free nutrient solution (Yu et al., 2016) which consisted of (l^−1^ Milli-Q H_2_O): KCl 0.5 g, CaSO_4_·2H_2_O 0.2 g, MgSO_4_·7H_2_O 0.2 g, KH_2_PO_4_ 0.2 g, Fe-EDTA 0.001 g, KNO_3_1 g, trace elements (containing H_3_BO_3_ 0.1 g, ZnSO_4_·7H_2_O 0.1 g, CuSO_4_·5H_2_O 0.05 g, MnCl_2_·4H_2_O 0.05 g and Na_2_MoO_4_·2H_2_O 0.01 g), and CaCO_3_ 2 g which was added to the final solution and stirred well. Vermiculite was used as substrate for the pot culture experiment with added metals. Stock solutions of CdCl_2_ and MnCl_2_ were separately prepared in Milli-Q water. Metal-spiked vermiculite was prepared by adding 190 ml of the stock solution to vermiculite (200 g dry wt) to achieve desired concentrations at 5, 20, and 35 mg kg^−1^ Cd or Mn in the substrate. The apparatus was assembled by filling the pot with vermiculite to 90% and autoclaved at 115°C for 30 min. Homogeneity was tested by measuring vermiculite samples randomly taken from at least five spots across different depths. Physico-chemical properties of V-Ti magnetite tailings and vermiculite were determined using standard methods (Pansu and Gautheyrou, 2007). Seeds of *L. leucocephala* were surface sterilized by soaking in 95% alcohol for 10 s and in 5% sodium hypochlorite solution for 5 min before they were rinsed with at least 10 changes of sterile Milli-Q water (Somasegaran and Hoben, 2012). After sterilization, the seeds were placed on plain agar (0.05%) for germination at 25°C in the dark. Three burgeoning seeds were aseptically transferred into each pot and inoculated with 1 ml rhizobial suspension at the root of each seedling. After this, a layer of 1 cm autoclaved quartz sand was placed on top of the vermiculite to ensure seedlings grew in a sterile environment. Each metal concentration had three pots as replication and non-inoculated plants served as the control group. The plants were grown in a greenhouse for a total length of 6 months at a day temperature of 25°C for 16 h and night temperature of 17°C for 8 h. Upon harvest, nodule number, plant height and root length were measured and freshly harvested samples of *L. leucocephala* were immediately oven-dried at 105°C for 30 min and maintained at 80°C until constant weight. Dried plant samples were pulverized for following experimentation.

### Determination of plant metal contents

Dried tissues of the leucaena plants were finely ground and digested with a concentrated mixture of HNO_3_ and HClO_4_ (5:1, v/v) prior to the measurement of heavy metal concentrations using ICP-AES (IRIS Intrepid II, Thermo Electron, USA). The remediation capabilities of *L. leucocephala*-rhizhobia system were determined by calculating bioconcentration coefficient factor (BCF) and translocation factor (TF), which were defined as follows: BCF = root metal content (mg kg^−1^) / soil metal content (mg kg^−1^); TF = shoot metal content (mg kg^−1^) / root metal content (mg kg^−1^).

### Determination of N, P, and K in plants

Plant samples were digested by sulphuric acid prior to the determination of nitrogen (N) using indophenol blue method, phosphorus (P) using vanadium molybdate method, and potassium (K) using a flame photometer (FP6410, Shanghai Precision & Scientific, China) (Novozamsky et al., 1974).

### Statistical analyses

Statistical analyses were done using the SPSS 22.0 package. Data were expressed as mean values ± standard deviations calculated using Microsoft Excel 2013. For the analyses of symbiotic effects and metal contents in plants, means between different metal treatments were compared using one-way analysis of variance (ANOVA) and least significant difference (LSD) test. Means between the inoculated and non-inoculated groups at each metal concentration were compared using Tukey's *post-hoc* test. Differences were considered statistically significant at *P* < 0.05.

## Results

### Metal contents of V-Ti magnetite tailing soil

Heavy metal assay revealed that the topsoil of V-Ti magnetite tailings at Panzhihua mainly contained 7 heavy metal elements (Table 1). Exceedingly high amounts of Fe (143.3 g kg^−1^) and Ti (35.7 g kg^−1^) were found. However, the concentration of V was only 0.3 g kg^−1^. Compared to Fe, Ti, and V, Mn (2.8 g kg^−1^), Cu (58.4 mg kg^−1^), Ni (92.3 mg kg^−1^), and Cd (7.1 mg kg^−1^) existed in moderate amounts.

**Table 1:** Heavy metal contents in the tailings and plant tissues and metal uptake and translocation in *L. leucocephala* after 6 months growth in V-Ti magnetite tailings.

**Samples**	**Metal content (mg kg^−1^)**						
	**Fe**	**Ti**	**V**	**Mn**	**Cd**	**Cu**	**Ni**
Tailings	143331.2 ± 1206.7	35681.0 ± 380.0	311.4 ± 19.0	2767.4 ± 37.0	7.1 ± 1.7	58.4 ± 3.9	92.3 ± 0.58
YH1-Root	3609.7 ± 47.1b (2.8%)	277.2 ± 17.3b (0.9%)	9.5 ± 0.6b (3.1%)	431.5 ± 7.5a (19.8%)	0.4 ± 0.02b (5.3%)	80.3 ± 8.6b (156.0%)	16.5 ± 1.1b (22.5%)
CK-Root	5110.9 ± 111.2a (4.2%)	729.1 ± 23.2a (2.4%)	16.0 ± 0.9a (5.2%)	452.6 ± 18.4a (21.6%)	1.1 ± 0.08a (15.5%)	166.1 ± 18.1a (329.4%)	35.6 ± 4.1a (48.8%)
YH1-Shoot	331.3 ± 6.9**b** (9.2%)	41.7 ± 7.8**b** (15.1%)	–	115.8 ± 9.5**b** (26.8%)	–	10.9 ± 2.7**b** (13.6%)	4.2 ± 1.5**b** (25.5%)
CK-Shoot	967.0 ± 30.6**a** (18.9%)	127.2 ± 16.4**a** (17.5%)	–	144.8 ± 20.7**a** (32.0%)	–	26.4 ± 5.1**a** (15.9%)	9.5 ± 2.5**a** (26.5%)

*The data are presented as mean values ± standard deviations of 3 replicates; normal letters indicate the comparison between values of YH1-Root and CK-Root while bold letters indicate the comparison between values of YH1-Shoot and CK-Shoot; “-” indicates data not applicable. Values from different treatments in each column are followed by different lowercase letters indicating statistical difference at P < 0.05 between inoculated and non-inoculated plants according to LSD test; Percentage values in bracket means the bioconcentration coefficient factor (BCF) of root and the translocation factor (TF) of shoot for metals; YH1-Root indicates the root of L. leucocephala inoculated with S. saheli YH1; CK-Root means the root of non-inoculated plants; YH1-Shoot indicates the shoot of L. leucocephala inoculated with S. saheli YH1; CK-Shoot means the shoot of non-inoculated plants*.

### Identification of isolate YH1

**Figure 2:**
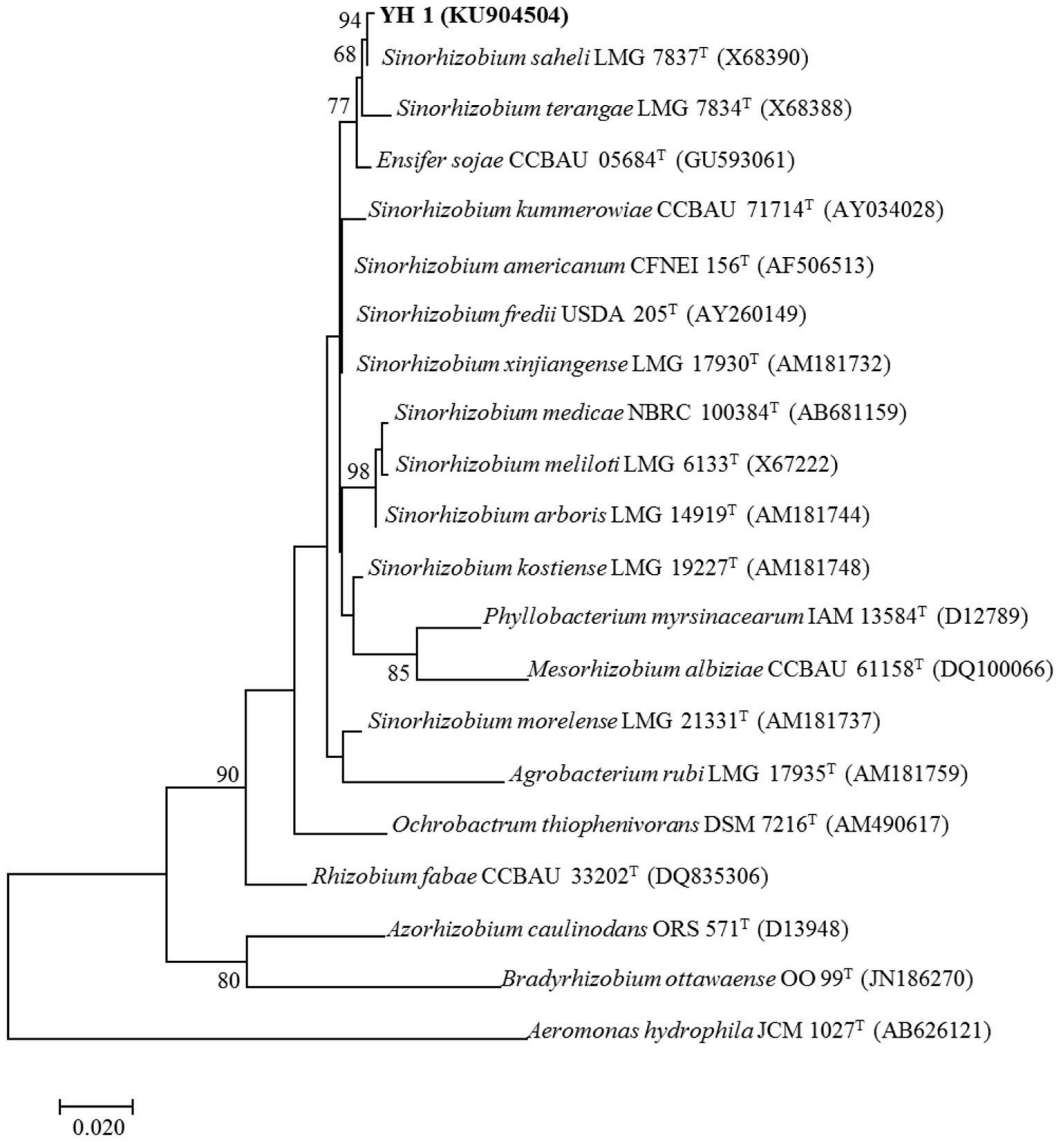
16S rRNA phylogenetic tree (1,404 bp) of *L. leucocephala*-isolated strain YH1 created using maximum likelihood method with the Kimura 2-parameter model. Bootstrap confidence level is based on 1,000 iterations. Accession numbers retrieved from GenBank are shown in parentheses. The scale bar implies 2% nucleotide substitutions per site. Values lower than 50 are not shown at the nodes. Superscript “T” following each strain number indicates type strain.

**Figure 3:**
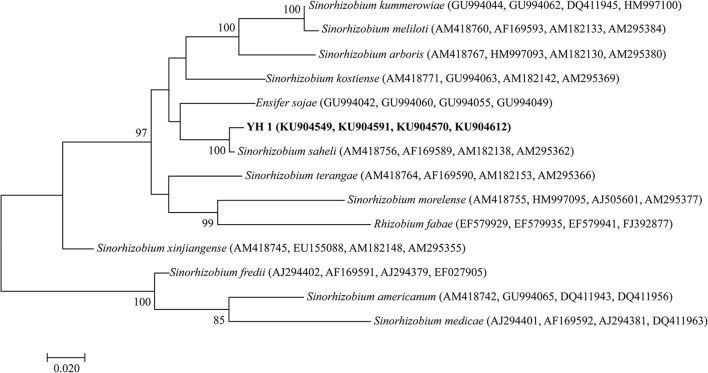
Concatenated tree of *L. leucocephala*-isolated strain YH1 combining *atpD-glnII-recA-rpoB* (1,631 bp), generated using maximum likelihood method (Kimura 2-parameter model) with 1,000 repetitions of bootstrap value. Accession numbers retrieved from GenBank are shown in parentheses. The scale bar denotes 2% nucleotide substitutions per site. Values lower than 50 are not shown at the nodes.

**Figure 4:**
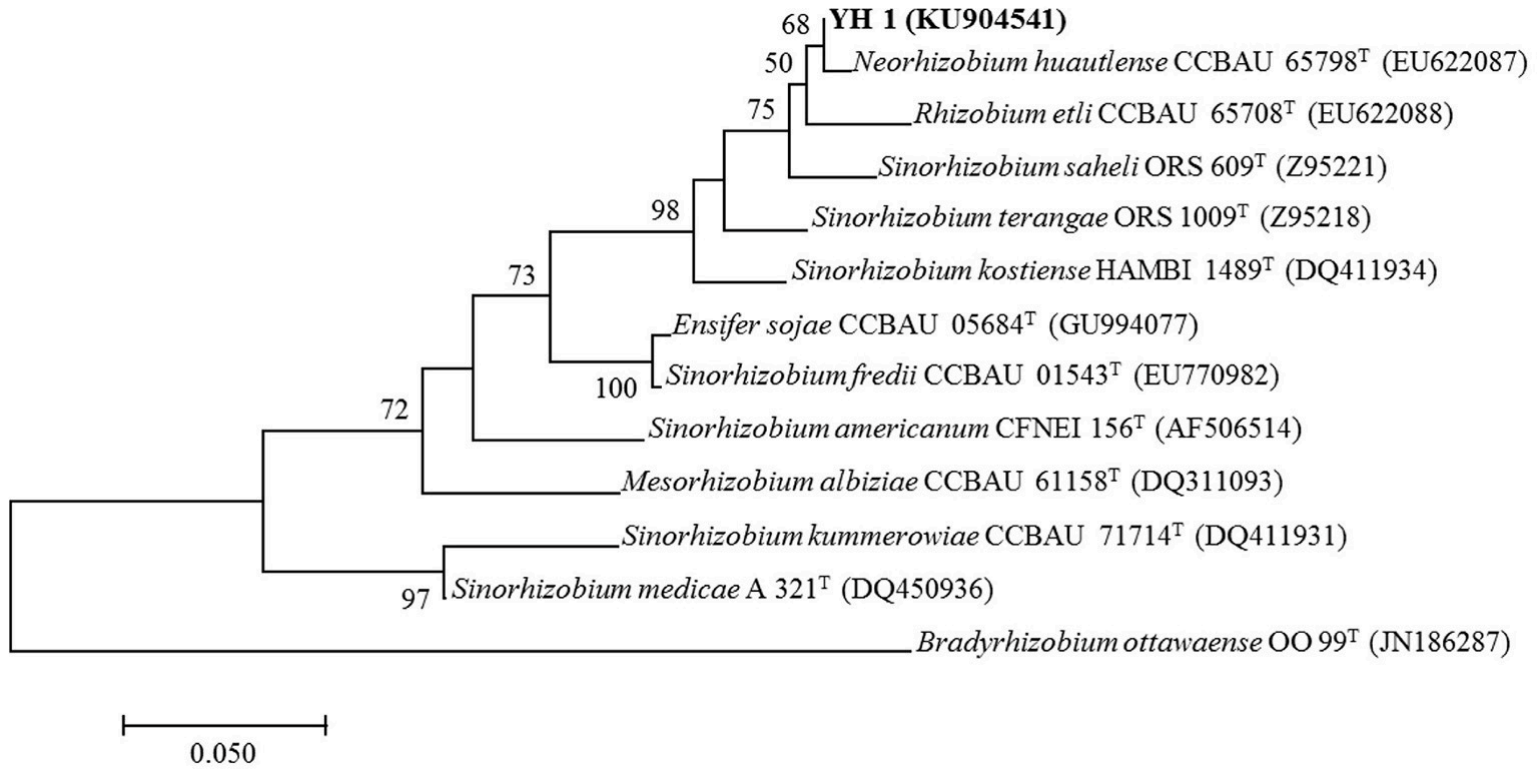
Maximum likelihood tree of *nifH* sequences (453 bp) for *L. leucocephala*-isolated strain YH1 constructed using the Kimura 2-parameter model with bootstrap confidence based on 1,000 repetitions. Accession numbers retrieved from GenBank are shown in parentheses. The scale bar denotes 5% nucleotide substitutions per site. Values lower than 50 are not shown at the nodes. Superscript “T” following each strain number indicates type strain.

All successfully amplified nucleotide sequences of isolate YH1 for phylogenetic analysis were uploaded to GenBank and accession numbers were obtained (KU904504, 16S rRNA; KU904541, *nifH*; KU904549*, atpD*; KU904591, *glnII*; KU904570, *recA*; KU904612, *rpoB*). The isolate was clustered into the same group with 99.9% similarity with *Sinorhizobium saheli* LMG7837 in the 16S rRNA phylogenetic tree (Figure 2). Therefore, it was preliminarily classified as belonging to *Sinorhizobium*. Moreover, the concatenated tree of the four housekeeping genes (*atpD*-*glnII*-*recA*-*rpoB*) assigned YH1 to the same branch with *Sinorhizobium saheli* with 99.0% homology (Figure 3). Therefore, isolate YH1 was identified as *Sinorhizobium saheli*. The nitrogenase reductase gene (*nifH*) tree which consisted of 12 other known species was constructed and it showed that the *nifH* gene of strain YH1 shared 99.0 % similarity with that of *Neorhizobium huautlense* (CCBAU 65798T) (Figure 4).

### Metal tolerance of YH1

Metal tolerance test revealed that vigorous growth of YH1 was observed at 50 mg kg^−1^ Cd and 250 mg kg^−1^ Mn, indicating its strong tolerance to these metals. Therefore, the metals of Cd and Mn were selected as metal contaminants for pot culture experiments to further explore the synergism of *S. saheli* YH1 with *L. leucocephala*.

### PGP traits of YH1

*S. saheli* YH1 secreted 166.77 ± 2.03 mg l^−1^ IAA in L-tryptophan-containing YMB medium after 3 days growth. An amount of 104.41 ± 7.48 mg l^−1^ soluble phosphate was detected in the supernatant of NBRIP after 7 days incubation. However, *S. saheli* YH1 did not show siderophore producing activity.

### Remediation effects in V-Ti magnetite tailings

The physico-chemical analysis showed that the tailing samples were at pH 6.66 ± 0.37 and contained 8.51 ± 0.43 mg kg^−1^ available nitrogen, 23.98 ± 2.33 mg kg^−1^ available phosphorus and 13.56 ± 1.11 mg kg^−1^ available potassium. *L. leucocephala* was nodulated by *S. saheli* YH1 in V-Ti mine tailings after 6 months growth, while no nodules were found on the roots in the control group. The nodule number was on average 38 for each inoculated plant. In general, the inoculation of YH1 led to 10.0% increase in plant nitrogen content and a significant increase (*P* < 0.05) in plant height and root length by 39.5 and 27.2% respectively (Table 2). There was also a significant (*P* < 0.05) increase of the plant biomass by 67.2% in comparison with the non-inoculated control. The contents of phosphorus and potassium were also significantly (*P* < 0.05) elevated by 112.2 and 25.0%, respectively (Table 2).

**Table 2:** Symbiotic nitrogen-fixing and PGP effects of strain YH1 on *L. leucocephala* after 6 months growth in V-Ti mine tailings and vermiculite supplemented with different concentrations of Cd or Mn.

**Pot soil**	**Treatment**	**Nodule number**	**Plant height (cm)**	**Root length (cm)**	**Total dry weight (g)**	**Total N (g kg^−1^)**	**Total P (g kg^−1^)**	**Total K (g kg^−1^)**
Tailings	YH1	32 ± 2ab	16.6 ± 0.7ab^*^	15.1 ± 2.5c	1.0 ± 0.2**a**	9.4 ± 0.1bc	1.8 ± 0.1ab^*^	18.5 ± 0.5a^*^
	CK	0	11.9 ± 0.9b	10.1 ± 2.3d	0.6 ± 0.2**a**	9.0 ± 1.0b	0.9 ± 0.1d	14.9 ± 1.2ab
Cd (5 mg kg^−1^)	YH1	23 ± 5bc	15.1 ± 2.2ab	23.6 ± 1.0ab^*^	0.3 ± 0.0c	8.2 ± 0.3bc	1.6 ± 0.2ab	15.7 ± 0.6ab
	CK	0	16.3 ± 2.8a	12.6 ± 0.7cd	0.3 ± 0.1c	8.2 ± 0.2b	1.1 ± 0.1cd	10.7 ± 1.8c
Cd (20 mg kg^−1^)	YH1	23 ± 5bc	12.7 ± 4.7b	23.8 ± 2.1ab^*^	0.5 ± 0.1bc	8.0 ± 0.8c	1.3 ± 0.1bc	15.4 ± 0.4ab
	CK	0	17.3 ± 0.9a	16.6 ± 1.7bc	0.4 ± 0.1abc	8.9 ± 0.1b	1.5 ± 0.3bc	14.2 ± 0.8ab
Cd (35 mg kg^−1^)	YH1	27 ± 5abc	15.7 ± 2.1ab	27.1 ± 2.9a^*^	0.5 ± 0.0bc	9.3 ± 0.8bc	1.2 ± 0.1bc	15.1 ± 2.5ab
	CK	0	17.3 ± 1.4**a**	16.2 ± 2.5*c*	0.4 ± 0.1bc	11.5 ± 0.8**a**	1.1 ± 0.1cd	12.0 ± 0.3bc
Mn (5 mg kg^−1^)	YH1	33 ± 5ab	19.6 ± 3.2ab	19.5 ± 1.5bc	0.6 ± 0.1bc	9.2 ± 0.8bc	1.6 ± 0.1b^*^	17.0 ± 1.7ab
	CK	0	16.7 ± 1.1a	21.0 ± 1.5ab	0.5 ± 0.0ab	8.8 ± 1.4b	0.9 ± 0.1d	14.0 ± 0.7ab
Mn (20 mg kg^−1^)	YH1	33 ± 9ab	17.2 ± 2.5ab	20.1 ± 3.4bc	0.7 ± 0.1b	8.0 ± 0.4c	1.2 ± 0.0bc	18.5 ± 0.0a
	CK	0	16.1 ± 0.7a	21.0 ± 1.6ab	0.6 ± 0.0a	7.8 ± 0.9b	1.6 ± 0.3b	16.3 ± 0.5**a**
Mn (35 mg kg^−1^)	YH1	17 ± 5c	15.1 ± 2.6ab	22.3 ± 3.2ab	0.5 ± 0.1bc	10.7 ± 1.6ab	0.5 ± 0.1c	14.3 ± 0.8b
	CK	0	14.5 ± 1.5ab	17.1 ± 3.3bc	0.3 ± 0.1c	9.6 ± 0.3ab	1.5 ± 0.1bc^*^	12.0 ± 0.9bc
NA (0)	YH1	40 ± 8**a**	21.7 ± 3.4**a**	22.0 ± 1.4ab	0.5 ± 0.1bc	12.2 ± 0.2a^*^	2.5 ± 0.8**a**	16.3 ± 0.7ab
	CK	0	15.7 ± 2.5a	23.1 ± 1.7**a**	0.5 ± 0.1abc	9.8 ± 0.0ab	2.2 ± 0.2**a**	13.4 ± 0.2bc

The data are presented as mean values ± standard deviations of three replicates. “CK” indicates the non-inoculated control plants. “NA (0)” denotes the plants in non-spiked vermiculite. Data in each column with different metal concentrations inoculated with the same strain (or without inoculation) are followed by different lowercase letters indicating statistical difference at P < 0.05 according to LSD test. The highest value in either the inoculated or non-inoculated group is expressed in bold lowercase letters. “

* *” is placed after the value that is higher the other with statistical significance (P < 0.05) between the inoculated and non-inoculated groups at each metal concentration according to Tukey's post-hoc test*.

The amounts of metal uptake in *L. leucocephala* roots from the V-Ti magnetite tailing soil are in the following order: Fe>Mn>Ti>Cu>Ni>V>Cd for inoculated plants and Fe>Ti>Mn>Cu>Ni>V>Cd for control group. Overall, the uptake of Cd was most while that of Mn was least effectively reduced. Comparing with inoculation-free plants, *S. saheli* YH1 significantly (*P* < 0.05) reduced metal uptake in roots, with the reduction rates for the tested metals ranging from 8.4 to 65.5%.

The translocation of these metals showed that only Fe, Ti, Mn, Cu, and Ni were transferred from roots to shoots. TF values for these transferred metals ranged from 9.2 to 26.8% for the inoculated plants and from 15.9 to 32.0% for the non-inoculated plants. The amount of Fe transferred was the highest among all metals tested in both inoculated and control groups, the former amounting to 331.3 mg kg^−1^ and the latter 967.0 mg kg^−1^. The inoculation of YH1 prevented these metals from moving to shoots, leading to a significant reduction (*P* < 0.05) of TF values for the inoculated plants. However, in the case of Fe, the inoculated plants exhibited less than 50% reduction in the amounts of the translocated metals compared with the control plants.

### Remediation effects in Cd/Mn soil

The non-spiked vermiculite was at pH 7.06 ± 0.04 and had the following properties: 56.02 ± 13.60 mg kg^−1^ total phosphorus, 0.67 ± 0.05 mg kg^−1^ available phosphorous, 15.75 ± 1.29 g kg^−1^ total potassium, and 75.80 ± 3.66 mg kg^−1^ available potassium. No nitrogen contents were detected for vermiculite.

#### Metal uptake by plants

**Figure 5:**
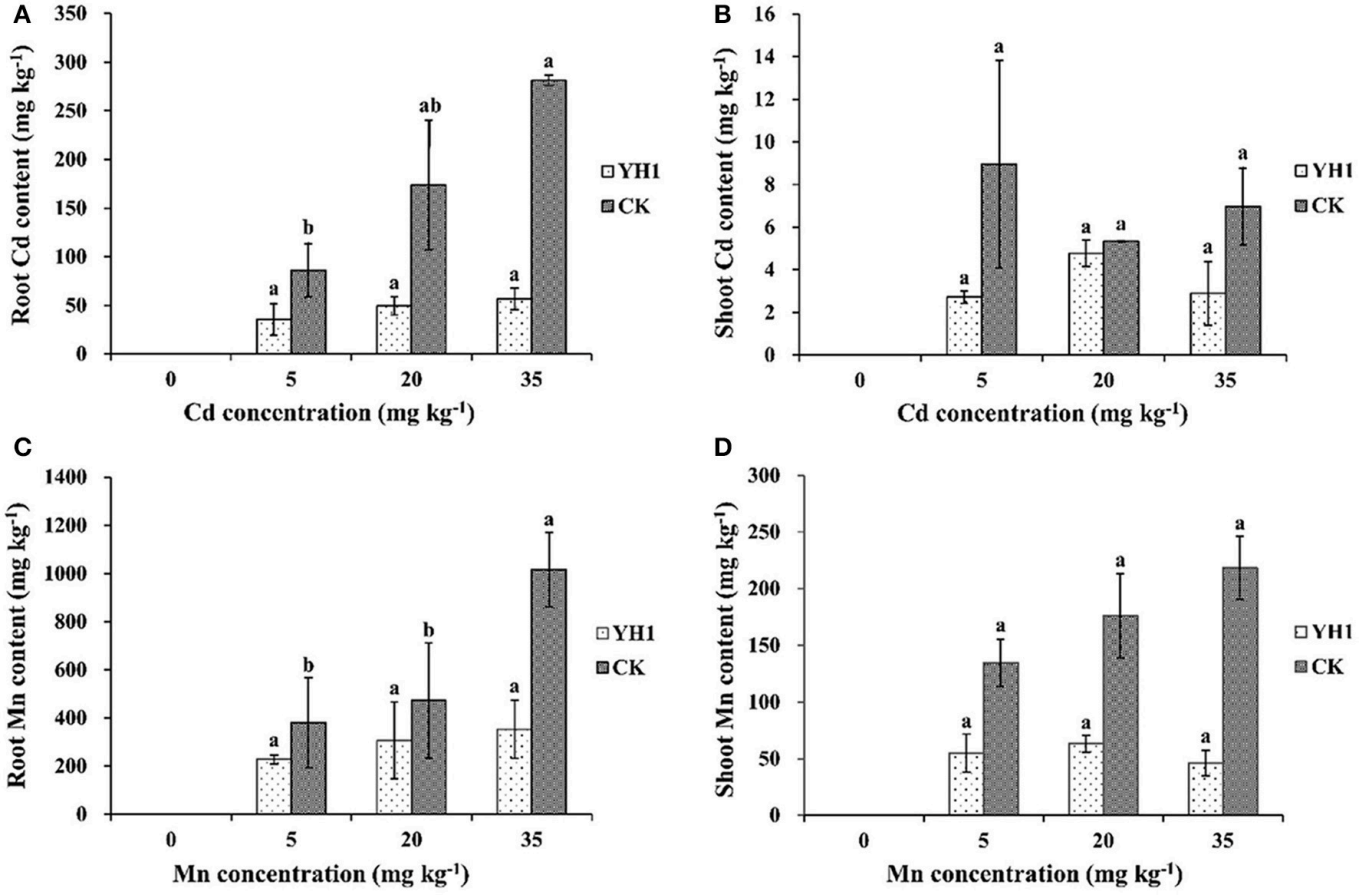
Cd contents in **(A)** shoot and **(B)** root, Mn contents in **(C)** root and **(D)** shoot of *L. leucocephala* after 6 months growth in vermiculite supplemented with three different concentrations of Cd or Mn. YH1, plants inoculated with strain YH1; CK, control group without inoculation. Different lowercase letters following data in either the YH1-inoculated or the YH1-free control group treated with different concentrations of the same metal show the statistical difference is significant at *P* < 0.05 according to LSD test. Error bars represent standard deviations.

In all plants inoculated with *L. leucocephala* grown in Cd- or Mn-amended soils, uptake of both metals by the plants was decreased compared to the control. Root uptake of Cd was positively correlated with the Cd content in control group, while it remained at a low level in all three inoculated treatments. The highest amount of Cd uptake was found in the soil added with 35 mg kg^−1^ Cd, where the roots of inoculation-free plants contained 281.33 ± 61.24 mg kg^−1^ Cd, 79.9% higher than in the YH1-inoculated group (56.43 ± 11.14 mg kg^−1^) (Figure 5A). However, shoot Cd contents in plants with YH1 inoculation were only slightly reduced (Figure 5B) compared to the control at all three soil Cd concentrations. There was no correlation of shoot Cd content to the soil Cd content in both groups. The highest amount of shoot Cd content in control group was 8.96 ± 4.87 mg kg^−1^ in the soil treated with 5 mg kg^−1^ Cd, while in inoculated group was 4.78 ± 0.61 mg kg^−1^ at 20 mg kg^−1^ soil Cd.

Root Mn contents in inoculated plants at all levels of soil Mn were also lower than in the control group (Figure 5C). In the presence of 35 mg kg^−1^ Mn in soil, the biological concentration factor (BCF) for Mn was reduced by 67.6% compared with the non-inoculated control. Shoot Mn contents in all inoculated plants were apparently reduced compared to the control, and in treatments with 20 and 35 mg kg^−1^ soil Mn, shoot Mn contents were significantly (*P* < 0.05) reduced by 64.1 and 78.9% respectively (Figure 5D).

#### Symbiosis and nitrogen fixation

All plants with inoculation of strain YH1 exhibited formation of root nodules. Less nodules were produced in plants treated with 5, 20, and 35 mg kg^−1^ cadmium than in those grown in Cd-free control. With the increasing amount of manganese in the background from 0 to 20 mg kg^−1^, the number of nodules was reduced (Table 2). However, more nodules were found in 35 mg kg^−1^ Cd soil. There was no significant difference between nodule numbers for plants grown in both metal-spiked soils at all concentrations.

#### Biomass yield

After growing for 6 months under greenhouse conditions, all inoculated groups saw a slightly improved biomass yield in the presence of both metals. Among all metal-treated plants, dry weight was increased with the inoculation of YH1, except for plants in 5 mg kg^−1^ Cd soil, where biomass remained the same for both groups (Table 2). However, the total dry weight of YH1-inoculated plants in 5, 20, and 35 mg kg^−1^ Cd soils did not show apparent difference compared to either inoculation-free or non-spiked control.

The biomass yield pattern for Mn-treated plants was different from that for Cd-treated plants. Dry weight of both inoculated plants and control showed positive correlation to Mn content ranging from 0 to 20 mg kg^−1^. However, in 35 mg kg^−1^ Mn soil, biomass yield of both groups decreased. YH1-free plants showed a significant decrease of biomass at 35 mg kg^−1^ soil Mn compared to all treatments with lower Mn contents (Table 2).

#### Plant height and root length

For inoculation-free plants in 0, 5, 20, and 35 mg kg^−1^ Cd soils (Table 2), no significant difference in plant height was found. In the inoculated group, plant height was reduced in the presence of different concentrations of Cd in soil. Only in non-spiked treatment, inoculated plants appeared to be higher than the control. YH1-inoculated plants showed a significant increase (*P* < 0.05) in root length by 86.9, 44.2 and 67.5% at 5, 20, and 35 mg kg^−1^ soil Cd respectively, except in the non-spiked control group, where YH1 did not contribute to the increase of root length. It showed that plant height was negatively correlated with the Mn content in soil for both YH1 and YH1-free groups. The introduction of YH1 led to a slight increase of plant height compared to plants without inoculation at 5, 20, and 30 mg kg^−1^ soil Mn (Table 2).

## Discussion

### Rhizobia identification

*L. leucocephala*-associated sinorhizobia in this region have been previously identified as nearest neighbors to *S. americanum, S. fredii, S. kummerowiae, S. meliloti, S. mexicanus, S. saheli*, and *S. xinjiangense* (Xu et al., 2013a,b). The existence of various toxic metals tends to exercise a natural selection process, through which metal-tolerant species are favored (Rajkumar et al., 2009). As was shown by our results, *L. leucocephala* was still able to grow vigorously and produce nitrogen-fixing nodules regardless of the presence of elevated amounts of toxic metals and the infertility in the V-Ti mine tailings, indicating the successful establishment of synergism between rhizobia and the host.

Both 16 rRNA and MLSA results identified strain YH1 as the closest neighbor to *Sinorhizobium saheli* (99.0% similarity), which is widely reported as a beneficial rhizobium to colonize *L*. *leucocephala* (Wang et al., 2006; Ardley, 2017). It is well known that the dinitrogenase reductase enzyme encoding *nifH* is accountable for the formation of root nodules with nitrogen-fixing capability (Laguerre et al., 2001). In our work, the symbiotic gene *nifH* of *Sinorhizobium* strain YH1 was clustered nearer to *Neorhizobium huautlense* (formerly known as *Rhizobium huautlense*) CCBAU 65798 with 99.6% homogeny than to *Sinorhizobium saheli* which only had 97.1% similarity. This could be explained by the fact that *N. huautlense* was also found to be a PGPR microbe that could reduce the accumulation of Cd by plant (Chen et al., 2016). In addition, a horizontal gene transfer could help further explain this phenomenon. It may be conjectured that the functional gene *nifH* of *Rhizobium huautlense* was accidentally obtained by YH1 due to close co-existence of the both species in the same region, as was proposed by other researchers before (Andrews et al., 2018).

### Pot culture experiment in tailings

Studies on the *in-situ* remediation of V-Ti tailings-polluted sites by the association of natively grown *L. leucocephala* and rhizobia are scarce. Mine tailings in Panzhihua region differ from other mine wastes due to their excessive amounts of extractable iron, titanium, and vanadium among other toxic metals.

*L. leucocephala* is not deemed as a hyper-accumulator as both biological concentration and translocation factors for most metals of interest were no more than 1.0. In spite of the higher concentrations of Fe and Ti in the tailings, the plants did not tend to absorb them as much as Cu and Ni, which are considered toxic to the plant at a lower dose. Phytoremediation of soils using legume-rhizobia associations can be generalized under two categories: mobilization, being the enhancement of metal uptake by the plants, owing to the production of various mobilizing agents such as biosurfactants, organic acids, siderophores and through biomethylation and redox effects (Ullah et al., 2015); immobilization or stabilization, the process in which the bioavailability of heavy metals in the rhizosphere is reduced due to on-root sorption and precipitation effects by root exudates and microbial metabolites (Salt et al., 1995; Wong, 2003). In this study, it may be concluded that *L. leucocephala*-YH1 symbiotic system, which led to 8.4% reduction of plant uptake for Mn and 65.5% for Cd in tailings, has the best reduction effect on Cd. This is again confirmed by vermiculite pot experiment where higher reduction rates on cadmium ranging from 59.7 to 79.4% were found. Consequently, the significant reduction (*P* < 0.05) of metal uptake except for Cd, as shown in the inoculated group probably implies the positive effects of the strain YH1 on the host which exhibit the immobilization feature in the root system.

Among a few leguminous tree species, *L. leucocephala* is able to tolerate higher concentrations of toxic metals compared to non-legumes and is less likely to succumb to multi-metal contaminated substrates (Chan et al., 1999). In addition, there is evidence showing the predominant status of this tree species in the topsoil of Pb/Zn mine tailings and the potential of using it as a phytoremediation tool (Zhang et al., 2001).

The growth state of plant-YH1 consortium demonstrated that, in contrast to control group, plants infected with strain YH1 showed successful nodulation and that biomass yield, plant height, root lengths and NPK contents were significantly elevated. This is indicative of the effectiveness of this inoculum and suggestive of the normal functioning of the symbiotic genes in it. It is widely reported that a number of PGPRs capable of increasing plant yield and improving soil conditions can be used in phytoremediation: these include *Achromobacter, Acinetobacter, Actinobacteria, Azotobacter, Bacillus, Flavobacterium, Ochrobactrum, Pseudomonas, Rhizobium*, and *Bradyrhizobium* (Reichman, 2007; Wani et al., 2008; Khan et al., 2009). For a long time, arbuscular mycorrhizal fungi (AMF) have been especially noted for improving phytoremediation by attenuating various metal stresses to the host apart from improving plant growth, whose mechanisms are frequently alluded to those of bacterial PGP strains (Lins et al., 2006). Discoveries of phytostabilization using rhizobia-legume systems came under notice when a number of rhizobial strains were reported to be both PGP-positive and capable of reducing metal uptake for the host. In a separate study, *Bradyrhizobium* sp. (*vigna*) RM8, isolated from green gram in metal contaminated sites, tolerant to high levels of nickel and zinc, active in promoting plant growth, was found to be able to cut nickel and zinc intake by the host while alleviating the toxic stresses (Wani et al., 2007). Another instance of phyto-immobilization was recorded by Dary et al. (2010), in which, an effective nitrogen fixer *Bradyrhizobium* sp. 750 reduced the accumulation of Cd, Cu, and Pb by *Lupinus luteus* in a field experiment on a multi-metal polluted site. There appears to be more metal enhancers than reducers and the former are often coupled with the ability of siderophore production (Glick, 2010), which may be attributed to the fact that siderophores as chelating agents make insoluble metal compounds bioavailable and thereby facilitating metal uptake. However, it works both ways, as established by Dimkpa et al. (2008), in which the accumulation of nickel in cowpea plants was lowered with the help of Ni-binding hydroxamate siderophores produced by *Streptomyces acidiscabies*, and this may prove that siderophores play a dual role in determining the uptake pattern regarding their various types while other more dominant factors may also have to be taken into account (Ma et al., 2011).

Phosphorus solubilization is another indispensable trait for soil microbes in the immobilization of heavy metals. Free metal ions can be readily precipitated as metal-P complexes of various mineral phases such, taking cadmium as an example, as Cd_5_H_2_(PO_4_)_4_·4H_2_O, Cd(H_2_PO_4_)_2_, Cd_3_(PO_4_)_2_ and amorphous cadmium phosphates at higher pH values (Sharma and Archana, 2016), which can be deposited on the surfaces of both roots and microbes in the rhizosphere resulting in reduced metal bioavailability and a reduction in both biological concentration and translocation effects (Park et al., 2011).

Indole-3-acetic acid is a phytohormone which has been widely regarded as an index for assessing the effectiveness of the promotion of cell elongation in plant tissues (Nadeem et al., 2015). The production of IAA by strain YH1 is higher (>100 μg ml^−1^) than most rhizobial stains previously reported and can be considered as an IAA-overproducer (Chiboub et al., 2016; Yu et al., 2017). It is observed that negative impact of metal accumulation inside plant tissues could be mitigated by the application of IAA (Nadeem et al., 2015). In our study of microbial phosphorus solubilization, strain YH1 was found to be more competent than most strains isolated from infertile and polluted soils, which among other PGP traits, further confirmed the effectiveness of strain YH1 to be potentially utilized in the phytoremediation of heavy metal contaminated soils.

As of today, there is limited information on the remediation of soils involving members from *Sinorhizobium*. It was revealed that some strains of *Sinorhizobium meliloti* helped with the uptake of Cd, Cu and Zn by *Medicago* plants with high translocation effects (Fan et al., 2011; Ghnaya et al., 2015; Zribi et al., 2015). Most interestingly, it was found that a symbiotic PGPR strain may help increase the uptake by one plant species while cause the decrease by another, as in the case of *Bradyrhizobium* sp. YL-6, where it boosted Cd uptake by *Lolium multiflorum* while reduced Cd uptake by *Glycine max* (Guo and Chi, 2013).

### Pot culture in Cd/Mn soils

Our work exhibited that the plants achieved greater biomass yield in both Cd and Mn tainted soils. In this experiment, the cross influence of other metals was minimized by using single metal-spiked vermiculite as substrate. Treatments with YH1 inoculum were all indicative of the successful formation of synergism with the microbe. By comparing plant dry weight between different treatments and the tolerance of YH1 to Cd and Mn, it is obvious that Cd appears to exercise more negative effects on both plant and rhizobia, and this may be explained by the fact that Cd has both higher microbial toxicity and phytotoxicity than Mn (Lambers et al., 2015; Ullah et al., 2015) and that Mn content is way higher in the tailings from which this strain was isolated. Biomass yield and growth parameters of leucaena plants were both reduced under the stress of cadmium even at a low concentration of 25 ppm as revealed by a previous study (Shafiq et al., 2010). Both Cd and Mn can stunt root growth and have damaging effects to leaves (Khan et al., 2011). It has been well explained that, several sophisticated microbial mechanisms conspire to curb the metal bioavailability to plants, which include biosorption onto the outer wall, intracellular sequestration, and complexation by certain biogenic anions (Gadd, 2004).

Root is where the toxic metals exert direct influence on the plant. Changes in this microbial-rhizospheric niche may alter the composition and patterns of exudation, which can further lead to damage to the root-hair cells. The significant increase in root length against the increasing amount of Cd, is probably attributed to the reduced metal stress caused by the immobilization effects.

There existed a great difference regarding the overall uptake of these two separately added metals, in which Mn uptake was more than three times the amounts of Cd in both groups and the translocation for Mn was more than 10 times that for Cd. Manganese exhibits extreme toxicity to plant cells in excessive amounts (>500 mg kg^−1^ in content) and is positively linked to soil acidity and a lack of other exchangeable metal ions such as Ca, Mg, and Fe in the rhizosphere (de Varennes et al., 2001). The reduction of Mn uptake by plants can be more complicated as common microorganisms are usually not directly involved in this process except for manganese oxidizing bacteria, which can increase Mn availability through the release of low weight bacteriogenic acids as IAA (Millaleo et al., 2010). Therefore, it may be conjectured that Mn resistant strain YH1 helped alleviate manganese uptake through indirect mechanisms by altering the plant exudation patterns, as suggested in studies on AMF-plant interactions (Nogueira and Cardoso, 2003), where plant exudates were changed under the influence of microbial activities resulting in an immobilization effect and reduced metal uptake by the plant.

Cadmium is a toxic metal with high mobility in both plant tissues and soils, the uptake of it by plants is in rise with the increase of its background concentration in both inoculated group and control. It is apparent that in metal-spiked soil, the uptake of Cd is also drastically reduced with the inoculation of YH1 especially under higher contents, and the plant translocation factor for Cd was also decreased with the inoculation of YH1, which is consistent with the tailings experiment and further indicates the metal-immobilizing effects on the legume.

### Nodulation under Cd or Mn stress

The decreasing trend in nodule yield under both metal stresses against the increasing levels of soil metal contents from 5 to 20 mg kg^−1^ was reversed in the treatment with 35 mg kg^−1^ Mn, which may be accounted for by the fact that manganese is less toxic to plants and its microbial symbionts. Manganese is an indispensable trace element constituting the reactive centers of various enzymes and is more than 20 times the amounts of the other metal pollutants in the V-Ti tailings. It should be noted that excessive ingestion of Mn can also cause toxicity to both plants and bacteria (Zornoza et al., 2010). Investigations by predecessors discovered that although Mn exists in plants in fairly large amounts, its toxicity can still affect the bacterial growth and legume-rhizobia associations (de Varennes et al., 2001; Hayes et al., 2012). However, our results confirmed that Cd appears to be more toxic than Mn, since the nodule number was lower than in plants treated with Mn at the same amounts of 5 and 35 mg kg^−1^.

## Conclusions

In conclusion, metalliferous V-Ti magnetite tailings from Panzhihua region harbor a PGP-positive rhizobia species, which was identified as *Sinorhizobium saheli* YH1. This strain exhibited IAA-producing and phosphate-solubilizing activities and was tolerant to high amounts of Cd and Mn. It also improved plant height, root length, and biomass yield for *L. Leucocephala* grown in both V-Ti tailings and soils amended with Cd/Mn. In particular, strain YH1 demonstrated abilities to nodulate the plant and reduce the uptake of heavy metals for the plant in the tailings and Cd- / Mn-supplemented soils. Our results thus provide further understanding of the efficiency of *S. saheli* YH1 in promoting plant health under heavy metal-ridden soil environments and suggest that it could be potentially used as an inoculum for the phytoremediation of metal-contaminated soils.

## Author contributions

XK and XY conceived the idea and carried out the experimentation. YZ, YL, QC, QW, and WT provided the funding. YC, YG, KZ, QX, and MM supervised the project. LZ, XZ, and LH analyzed the data. JK offered language polishing and editing service.

### Conflict of interest statement

The authors declare that the research was conducted in the absence of any commercial or financial relationships that could be construed as a potential conflict of interest.
