# Supplementary material for: Inoculation of Sinorhizobium saheli YH1 Leads to Reduced Metal Uptake for Leucaena leucocephala Grown in Mine Tailings and Metal-Polluted Soils
Source: Front Microbiol. 2018 Aug 27;9:1853. doi: 10.3389/fmicb.2018.01853 (PMC6119820; doi:10.3389/fmicb.2018.01853)
Supplement: Supplementary file 1 [file Image_1.pdf]

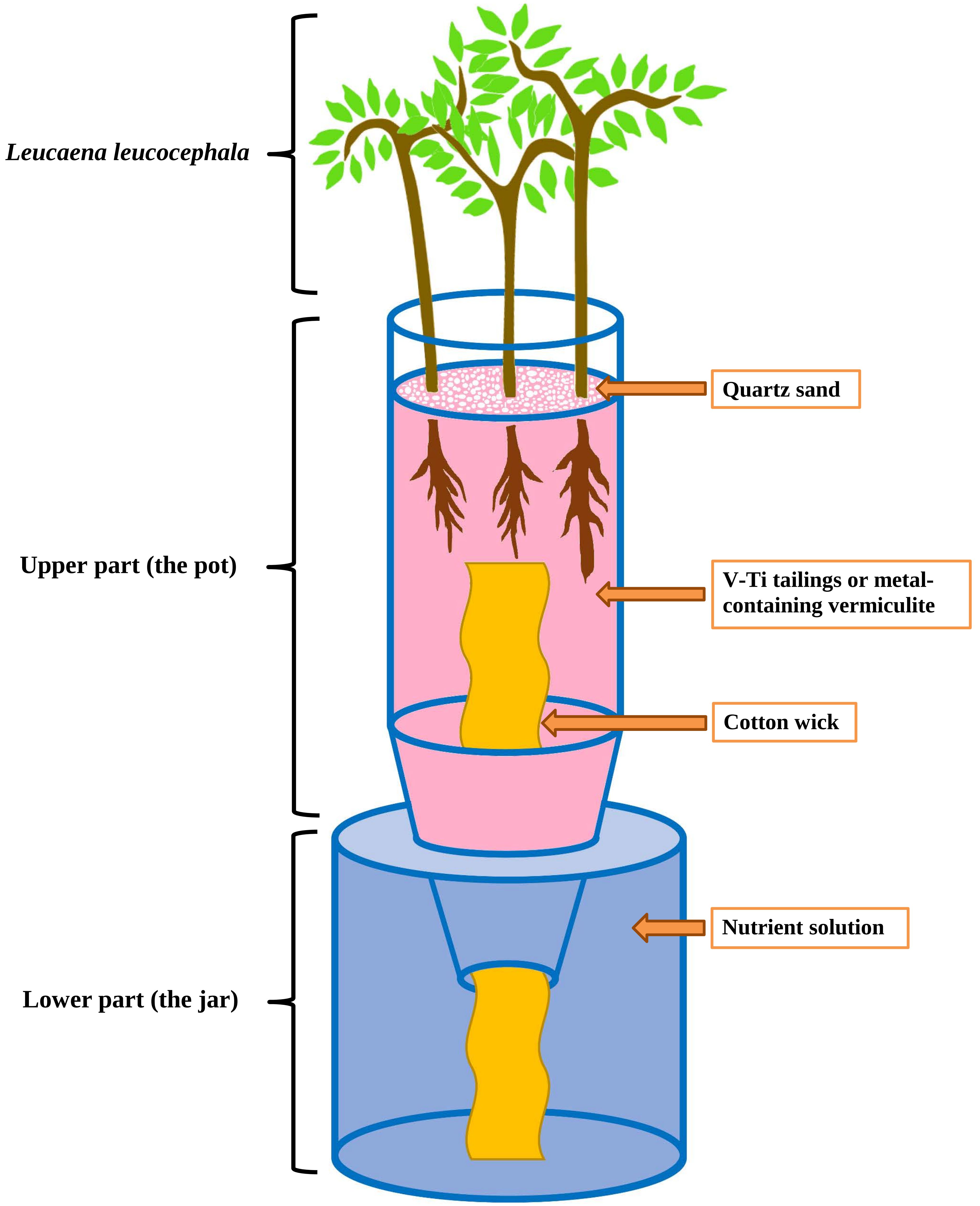

Figure S1. The modified Leonard's jar assembly used in the pot culture experiment. Seedlings of *Leucaena leucocephala* were planted in the V-Ti magnetite tailings or metal-spiked vermiculite contained in the upper part pot. The nutrient solution was stored in the lower part jar. These two parts were connected with a cotton wick which ensured the plants access to the nutrients. The topsoil was covered with a layer of 1 cm sterile quartz sand to prevent microbial contaminations from the air.
